# Applications of Bioadhesives: A Mini Review

**DOI:** 10.3389/fbioe.2021.716035

**Published:** 2021-09-03

**Authors:** Wanglin Duan, Xiangbing Bian, Yazhong Bu

**Affiliations:** ^1^ Department of Biophysics, School of Basic Medical Sciences, Health Science Center, Institute of Medical Engineering, Xi’an Jiaotong University, Xi’an, China; ^2^ The First Medical Center of Chinese PLA General Hospital, Beijing, China

**Keywords:** bioadhesive, sealant, wound closure, functional wound dressing, medical device fixation

## Abstract

Bioadhesives have demonstrated their superiority in clinical applications as tissue adhesives, hemostats, and tissue sealants. Because of the intrinsic stickiness, the applications have been expanded to various areas, such as functional wound dressing, factor delivery vehicles, and even medical device fixation. While many literature works discussed the mechanism of bioadhesives, few of them specifically summarized the applications of bioadhesives. To fill in the blanks, this review covers recent research articles and focuses precisely on the applications of bioadhesives which can be generally classified as follows: 1) wound closure, 2) sealing leakage, and 3) immobilization, including those already in the clinic and those showing great potential in the clinic. It is expected that this article will provide a whole picture on bioadhesives’ applications and lead to innovations in the application of bioadhesives in new fields.

## Introduction

Bioadhesives have been changing the surgical process with increasing importance and rapid development over the past 30 years (Ge and Chen, 2020; Taboada et al., 2020). The growing interest in producing adhesives and sealants makes them constitute a market share of $38 billion currently (Spotnitz and Burks, 2012; Qu et al., 2018; Liang et al., 2019). Compared with traditional invasive wound closure methods, including sutures, wires, and staples, bioadhesives have less possibility to damage the tissues and can promote wound healing through different mechanisms. For example, the bioadhesives possess antibacterial, anti-inflammatory, and antioxidant properties (Giano et al., 2014; Zhao et al., 2020). Other properties like self-healing and injectability significantly increase bioadhesives’ ease of use (Sun et al., 2020). Preventing leakage is also an essential role of bioadhesives. Leakage happens easily after the surgical process, which is up to 30% in some challenging situations. The leakage will easily lead to pain, inflammation, infection, and a high mortality rate (Artzi, 2013; Slieker et al., 2013; Pausch et al., 2020). With an aim to prevent those postoperative leakages, different bioadhesives have been developed accordingly. FocalSeal® was developed to avoid air leakage during lung surgery. DuraSeal® was designed for the spine and dura sealing. Coseal® was used as an adjunct of suture to prevent the leakage of blood vessels.

Moreover, they can remain stable on the site of application because of the intrinsic adhesion property. So, another important function of bioadhesives is immobilization. They can immobilize themselves as functional wound dressings to promote wound healing without other fixation methods (Yang et al., 2021). They can also be employed as vehicles to deliver functional items like drugs or cells to realize local delivery (Patel et al., 2014; Hu et al., 2021). With the development of smart biomedical devices, like wearable devices, implantable detectors, or sensors, a question has been raised about how to fix those devices on/in the body through noninvasive methods without damaging the tissues or the medical devices, to which the bioadhesive is also a good solution (Hwang et al., 2018; Deng et al., 2021).

**FIGURE 1:**
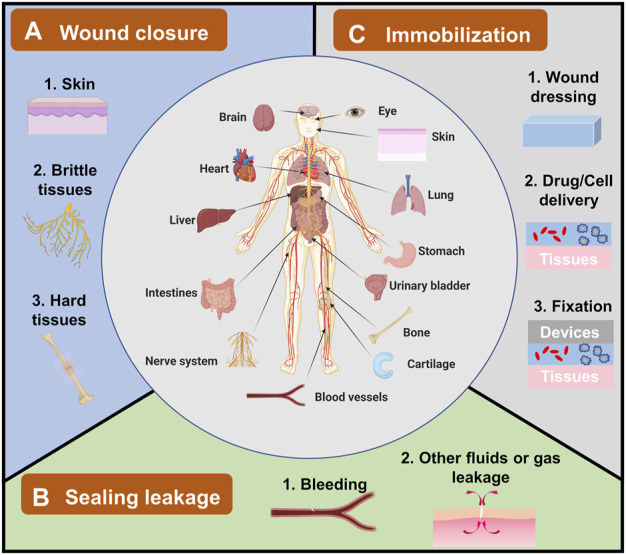
The applications of bioadhesives in human bodies and their categories. Bioadhesives have been explored in human bodies in different organs, including the brain, eyes, heart, liver, and skin. Their applications can be grouped into three categories. **(A)** Wound closure, which has been used in topical skin, and brittle/hard tissues. **(B)** Sealing leakage, including most explored blood leakage and other fluids or gas leakage. **(C)** Immobilization for wound dressing, drug/cell delivery, and fixation of devices.

Since bioadhesives are being explored in all sorts of fields, there is a need to summarize these applications, including the existing ones and potential ones. However, till now, most reviews focused on either bioadhesives’ adhesion mechanism or their applications on wound closure and leakage prevention; they seldom specifically discussed the overall applications of bioadhesives (Zhu et al., 2018; Bao et al., 2020; Ge and Chen, 2020; Taboada et al., 2020). Hence, in this review, the applications of bioadhesives have been summarized and grouped into three categories (Figure 1): 1) wound closure, 2) sealing leakage, and 3) immobilization. The examples of each category were demonstrated with the hope of providing a whole picture of the applications of bioadhesives and accelerating the innovation of bioadhesives in new fields. It is worthy to note that some bioadhesives own properties of two or three categories. Here, the bioadhesives are grouped according to their primary functions and the authors’ understanding of the bioadhesives.

## Wound Closure

**FIGURE 2:**
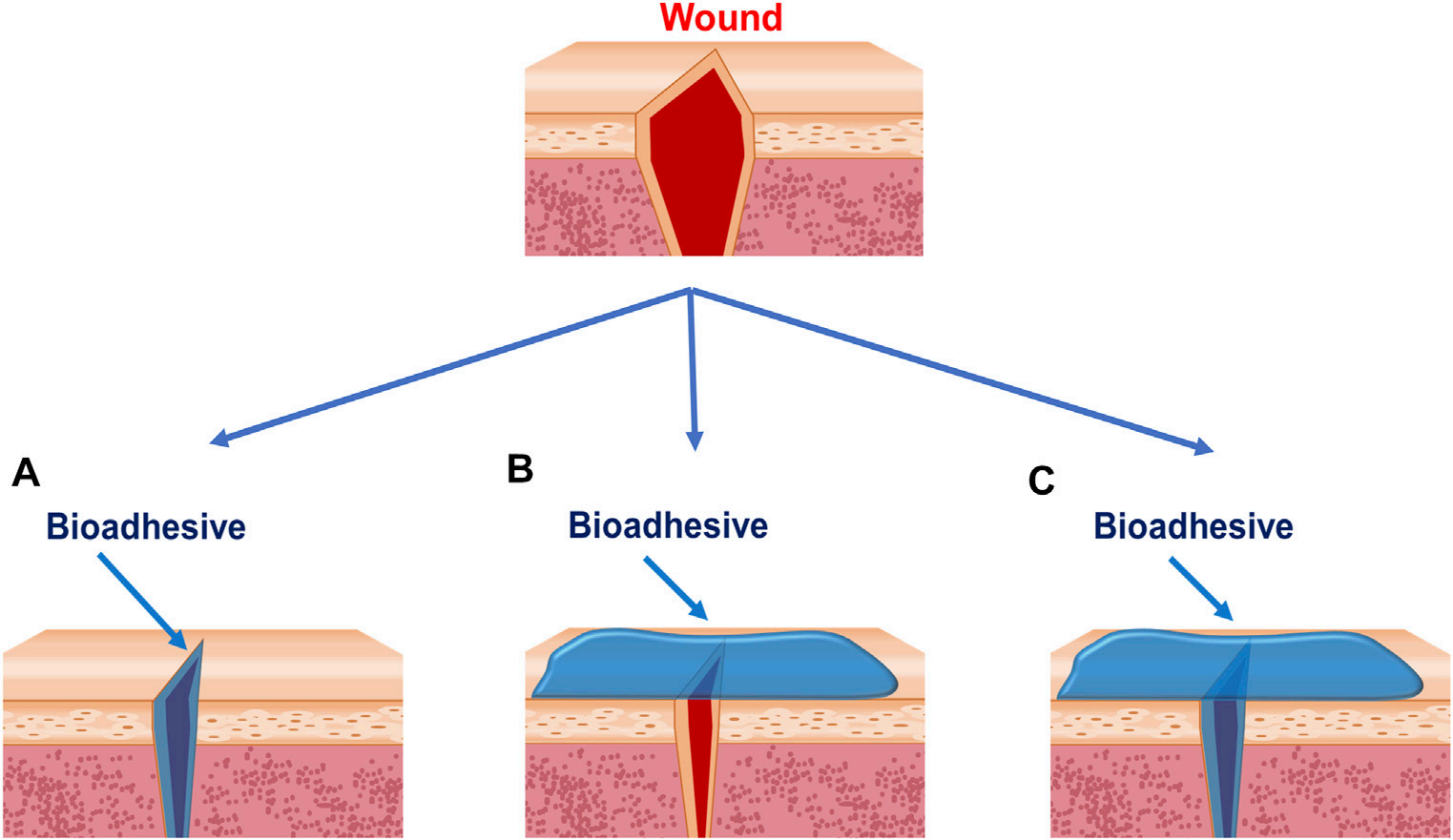
Strategies bioadhesives used to close wounds. **(A)** Bioadhesives are applied between wound edges. **(B)** Bioadhesives are applied outside of the wounds. **(C)** Bioadhesives are applied between and outside the wounds.

Wound closure is one of the most widely used applications of bioadhesives (Table 1). Sutures, wires, and staples have been the routine practice of wound closure for many years (Mehdizadeh and Yang, 2013). However, concerns about the scar tissues, secondary injury, foreign body reaction, wicking-induced infection, impaired wound healing process, and complex postoperative care are still waiting to be addressed (Harsha and Vasudha, 2018). As a good alternative, bioadhesives can adhere two wounds together through a noninvasive behavior. Typically, bioadhesives close the wounds by three methods： bringing the two sides of an injury together from the wound surface (Figure 2A), bringing the tissues beneath the surface together (Figure 2B), or closing wounds in both ways (Figure 2C). Firm adhesion is the property needed for all the three types. Moreover, the bioadhesives applied to wounds (Figures 2A,C) should be biodegradable and biocompatible and should not hinder the healing process (Li et al., 2020). The bioadhesives used on the surface are generally tape-like ones with strong cohesion strength (Bae et al., 2013; Yang et al., 2013). Cohesion, which is defined as the internal strength of an adhesive, together with adhesion creates a strong bond; few people conducted in-depth research on cohesion strength alone. However, it is reported that the photo-crosslinking strategy is commonly used to develop tape-like bioadhesives with high cohesion strength. Besides, the double network strategy has also been used to develop bioadhesive tapes with good wound closure efficacy by increasing the cohesion strength (Liu et al., 2018; Yuk et al., 2019; Pausch et al., 2020). Cyanoacrylate-based bioadhesives are the most widely used tissue adhesives for wound closure in the market, initially synthesized in 1949 (Harsha and Vasudha, 2018). Although they are not tape-like, they play roles mainly according to the second type because of their strong adhesion strength. However, their applications on wet tissues were limited because of their water-initiated curing. They also raise security concerns for exothermic polymerization, cytotoxic degradation products, and long degradation time (Bu et al., 2017; Harsha and Vasudha, 2018). So, special attention should be paid to avoid pushing cyanoacrylate-based bioadhesives into the wound, which can cause irritation and foreign body reaction. There are indications of holding wound edges together for at least 30 s before releasing. Because of the brittle property of the barriers formed by cyanoacrylate, it is suggested that cyanoacrylates are not suitable for wounds over joints, like the knees, groins, or hands, where adhesion easily fails because of the skin torsion (Harsha and Vasudha., 2018).

**TABLE 1:** Bioadhesives for wound closure.

Materials used	Type of the model	Animal species	References
Skin closure			
N-acryloyl, 2-glycine (ACG), hydroxyapatite (HAp)	Three incisions (2 cm) were cut on the back of rats	Male SD rats	Cui et al. (2018)
Secretion of *Andrias davidianus* (SSAD)	Four incisions (2 cm) were made for each rat	Male SD rats	Deng et al. (2019)
Eight-arm poly (ethylene glycol), tannic acid (TA)	Two incisions (1.5 cm) were made in the separate lateral ribs at the same distance from the rats’ midline	Female SD rats	Sun et al. (2020)
Poly (ethylene glycol) diacrylate, quaternized chitosan, tannic acid	Two full-thickness skin incisions (2 cm) were made on the rat’s back	Male BALB/c mice	Du et al. (2019)
Chitosan–poly (ethylene glycol)–tyramine (CPT)	Skin incisions (1.5 cm) were made on both sides of the rat’s back	Normal SD rats	Lih et al. (2012)
Deacetylated carboxymethyl chitin, N-acetylated carboxymethyl chitin	A full-thickness incisional wound (1 cm) was created on the rats	Wister rats	Azuma et al. (2015)
Citric acid, poly (ethylene glycol), dopamine	Six full-thickness wounds (2 cm long × 0.5 cm deep) were made on the dorsum of each rat	Female SD rats	Mehdizadeh et al. (2012)
Hydrophobic T8 polyhedral oligomeric silsesquioxane (POSS), trifluoromethanesulfonic acid	Skin incisions (1 cm in length and 1 cm in depth) were created on both sides of the pig’s back	Bama miniature pigs	Bu et al. (2017)
Mussel adhesive proteins (MAPs)	Skin incisions (2 cm) were made on the skin of the back	Normal SD rats	Jeon et al. (2015)
Tannic acid (TA), gelatin methacrylate (GelMA)	The tension incision model was made by removing a piece of olivary full-thickness skin around 1 cm in length	Athymic mice	Liu et al. (2018)
4-Arm polyethylene glycol propionaldehyde (PEG-PALD), chitosan (CS)	Two linear, full-thickness surgical wounds (1.3 cm) were created on both sides of the spine	Balb/c mice	Li et al. (2020)
Comminuted fracture			
Citric acid, poly (ethylene glycol), dopamine, hydroxyapatite (HA)	Osteotomy was performed at two sites with a surgical electric saw to produce a 10-mm length bone block. The bone blocks were cut into several segments (usually 3–4 fragments) using a bone rongeur	Male New Zealand Rabbits	Xie et al. (2015)
Chitosan, glycerol, glutaraldehyde	Bone sheets were cut into rectangular cuboids with a constant transversal section of 13 mm × 7.0 mm. Along 30 mm in the cuboid center, the transversal section was reduced to 6.5 mm × 6.5 mm. Subsequently, 4-mm diameter holes were drilled at both ends of the cuboid. Finally, the drilled cuboid was cut in half at the center of its longitudinal axis with a diamond wheel	Cancellous bones extracted from bovine humerus head	Cedano Serrano et al. (2017)
Nerve injury closure			
Octa-arm poly (ethylene glycol), octa-arm PEG-amine, LiCl	The sciatic nerve in the right leg was subjected to a transection at 0.5 cm distal to the sciatic notch, then octa-PEG-SS and LiCl-octa-PEG-NH2 were injected into the interface of the proximal stump and distal stump of the transected nerve	SD rats	Bu et al. (2020)
Vascular anastomosis			
Poloxamer, 2-octylcyanoacrylate adhesive (Syneture)	The left common iliac artery of the rat was divided and ligated at the bifurcation and anastomosed to the right common iliac artery in an end-to-side fashion. Then 2-octylcyanoacrylate was applied in a circumferential manner to complete the anastomosis	Male Fisher rats	Chang et al. (2011)

### Skin Closure

Skin closure is one of the main goals for wound closure-targeting bioadhesives which is in high demand because of the increasing workload of general surgery (Lu et al., 2020). This application has expanded popularity also because people pay more attention to their physical appearance. Dermal surgeons prefer using bioadhesives to improve their work efficiency. Patients tend to use noninvasive methods because there is usually less pain and a better cosmetic outcome (Ge and Chen, 2020). Luo et al. developed a new bioadhesive from the skin secretion of Chinese giant salamander. Later, the ability to close the wound was tested on the back of the rats with four incisions (2 cm). At the 5th day of postoperation, the bioadhesive-treated group showed the best healing effect among all groups, with no scar formation, infection, and inflammation (Deng et al., 2019). Du et al. fabricated an adhesive patch with poly-(ethylene glycol) diacrylate/quaternized chitosan/tannic acid based on mussel-inspired chemistry. The efficacy of the wound closure was tested on a full-thickness incision model. It was proved that at day 7 postsurgery, the patch-closed skin incisions exhibited more complete epidermis and dermis structures, and higher collagen deposition levels than the untreated tissues (Du et al., 2019).

### Wound Closure of Hard or Brittle Tissues

Other kinds of wound closure, in which bioadhesives show super advantages, are closing wounds of hard and extremely brittle tissues. In hard tissues like bones, bioadhesives provide a quick and straightforward method to fix the broken pieces, especially for small bone fragments (Farrar, 2012). For example, comminuted bone fracture is a severe orthopedic condition. The difficulty in fixation of the small bone pieces often leads to bone reduction, further resulting in bone displacement, bone union deformation, and nonunion. Based on citrate, Xie et al. developed an injectable bioadhesive to fix small bone pieces in comminuted bone fractures (Xie et al., 2015). Hydroxyapatite was added to the system to improve the healing efficacy. It was demonstrated that the bioadhesive increased bone formation with markedly enhanced three-point bending strength compared with the negative control. In extremely brittle or sensitive tissues like nerves, traditional sutures can cause irreversible damage. Besides, skilled surgeons are required for suturing those tissues, which entails prolonged surgical time and surgical skills. In our previous work, the octa-PEG–based bioadhesives have been used to close the nerve transection. After adding lithium chloride, the adhesive-reconnected nerves showed a low level of fibrosis, inflammation, and myoatrophy, as well as robust axonal regeneration and functional recovery (Bu et al., 2020). Corneal is another brittle tissue in which closure can be achieved by the bioadhesives. Shirzaei Sani et al. had engineered a gelatin-based adhesive biomaterial GelCORE to close the eye incision in an *ex vivo* model. It was found that the mean leak pressure of glue was more significant than that of commercial control groups (Shirzaei Sani et al., 2019).

## Sealing Leakage

Leakage is a common complication of surgeries and injuries. After lung resections, the incidence of air leakage was reported to be around 50% (Mueller and Marzluf, 2014). Cerebrospinal fluid leakage, caused by injuries or brain and sinus surgery, can lead to headaches, meningitis, and seizures. Gastric fluid leakage can cause severe tissue damage and infection, which happens easily after surgical procedures. So leakage prevention is vital in reducing operative risks, and decreasing the complications and the cost. Bioadhesives for leakage prevention are also called tissue sealants, which attracted the attention of researchers and have shown great potential in the clinic (Ryu et al., 2011; Nie et al., 2013; Behrens et al., 2014; Chan Choi et al., 2014; Kim et al., 2015; Chen et al., 2017; Yan et al., 2018; Kim et al., 2020). Some examples of the tissue sealants are summarized in table 2.

**TABLE 2:** Bioadhesives for sealing leakage.

Materials used	Type of the model	Animal species	References
Hemostasis			
Multi-vinyl monomers, dopamine	The rat femoral artery was punctured with a 1-ml syringe needle	Male SD rats	Cui et al. (2019)
Multi-vinyl monomers, dopamine	One-quarter of the liver lobe was sheared off	Male SD rats	Cui et al. (2019)
Poly (ethylene glycol), tyramine, chitosan; bovine serum albumin (BSA), Citrate acid, dopamine; chitosan, Pluronic®F127 (PF127-CHO)	Liver bleeding was triggered by puncture with a 20-G needle	SD rats; C57BL/6 mice; Kunming mice	(Lih et al. 2012, Zhu et al. 2017, and Qu et al. 2018)
Tannic acid, poly (ethylene glycol); chitosan/pluronic composite hydrogel; chitosan/poly-lysine hydrogel; poly (γ-glutamic acid), dopamine hydrochloride (DA); N-(3-aminopropyl) methacrylamide (APM); DOPA-modified gelatin; hydrazide-modified poly (L-glutamic acid) (PLGA–ADH), dual-functionalized alginate; epigallocatechin gallates (EGCGs), tyramine, hyaluronic acids, tyrosinase	Liver bleeding was triggered by puncture with an 18-G needle	Normal ICR mice; SD rats; Female balb-c mouse	(Ryu et al. 2011, Nie et al. 2013, Behrens et al. 2014, Chan Choi et al. 2014, Kim et al. 2015, Chen et al. 2017, Yan et al. 2018, and Kim et al. 2020)
DNA from salmon testes, tannic acid	Liver bleeding was triggered by puncture with a 23-G needle	ICR mice	Shin et al. (2015)
Glycol chitosan (GC), 3-(4-hydroxyphenyl) propionic acid	Liver bleeding was triggered by puncture with a 28-G needle	Male BALB/c mice	Lu et al. (2018)
Tetra-armed poly (ethylene glycol) amine, tetra-armed poly (ethylene glycol) succinimidyl succinate	An incision with a length to be 20 mm and a depth of 5 mm was made on the left lobe of the liver	New Zealand white rabbits	Bu et al. (2019)
Tetra-armed poly (ethylene glycol) amine, tetra-armed poly (ethylene glycol) succinimidyl succinate	A wound with a diameter of 25 mm and a depth of 10 mm was made on the spleen	Bama miniature pigs	Bu et al. (2019)
TachoSil (fibrinogen-impregnated sealant), TissuFleece and Tissucol Duo (fibrin glue)	A standardized left hemihepatectomy was performed by resecting the left and medial segment of the liver	Landrace pigs	Fonouni et al. (2017)
Chitosan	The femoral vein was transected	Male Long-Evans rats	Dowling et al. (2011)
Chitosan	The femoral artery was transected	Yorkshire crossbred swine,	Dowling et al. (2011)
N-(3-aminopropyl) methacrylamide hydrochloride (APM)	Tail amputation at 50% tail length was completed using surgical scissors	SD rats	Behrens et al. (2014)
Gelatin (Type A), methacrylic anhydride, polyethylene glycol diacrylate (PEGDA—Mn 700)	Rat tails were marked 4 cm from the tip and transected with a scalpel	Male Wistar rats	Krishnadoss et al. (2019)
N-(3-aminopropyl) methacrylamide hydrochloride (APM)	An incision of 5 cm in length and 1 cm in depth was made with a surgical scalpel on the right lobe of the sheep’s liver	Adult Dorsett hybrid sheep	Behrens et al. (2014)
Carboxymethyl chitosan (CMC), gelatin, oxidized alginate (OSA)	A wound about 1 cm in length and 2 mm in depth was made in one lobe of the liver	Normal SD rats	Cao et al. (2019)
Methacrylated gelatin (GelMA), N-(2-aminoethyl)-4-(4-(hydroxymethyl)-2-methoxy-5-nitrosophenoxy) butanamide (NB), glycosaminoglycan hyaluronic acid, lithium phenyl-2,4,6-trimethylbenzoylphosphinate	A 6-mm inner diameter needle was used to pierce the ventriculus sinister of pig hearts; an incision (4–5 mm) was created by needle puncture in the femoral artery	Male Bama Miniature pigs	Hong et al. (2019)
Methacrylated gelatin (GelMA), N-(2-aminoethyl)-4-(4-(hydroxymethyl)-2-methoxy-5-nitrosophenoxy) butanamide (NB), glycosaminoglycan hyaluronic acid, lithium phenyl-2,4,6-trimethylbenzoylphosphinate	A large (3 cm) incision was made in the liver; an incision (2 mm) was created in the femoral artery	Male New Zealand white rabbits	Hong et al. (2019)
4-Arm poly (ethylene glycol), 4-Arm poly (ethylene glycol) succinimidyl, 4-Arm poly (ethylene glycol) amine, vancomycin	An incision of 1 cm in length and 0.5 cm in depth was made in the liver	New Zealand white rabbits	Bu et al. (2016)
4-Arm poly (ethylene glycol), 4-Arm poly (ethylene glycol) succinimidyl, 4-Arm poly (ethylene glycol) amine, vancomycin	Femoral artery transection	New Zealand white rabbits	Bu et al. (2016)
Chitosan hydrochloride (ChitHCl), dextran dialdehyde (DDA)	Liver lobe edge resection of approximately 1.5 cm length at two sites; liver lobe circular excision of approximately 1 cm diameter at one site	New Zealand white rabbits	Balakrishnan et al. (2017)
Dextran sodium periodate	An incision of ∼1 cm in length and ∼0.2 cm in depth was fabricated with a surgical scalpel on the ear-vein of the rabbit; the uncontrolled hemorrhage model was created by cutting a wound on the rabbit’s femoral artery by using ophthalmic scissors	Male New Zealand white rabbits	Liu et al. (2019)
4-Arm-poly (ethylene glycol) succinimidyl, Lysozyme	The iatrogenic injury of the blood vessel was created by a 0.5 × 20-mm medical needle	Rabbits	Tan et al. (2019)
Glycerol, sebacic acid	Carotid artery defects model	Yorkshire pigs	Lang et al. (2014)
Other leakage prevention			
Bovine serum albumin (BSA), citrate acid, dopamine; gelatin, dopamine, genipin	Rat mastectomy model	Female SD rats	(Zhu et al., 2017 and Yanagihara et al. 2021)
Gelatin type A, methacrylic anhydride (MA), tannic acid (TA)	An incision (∼1 cm) was made on the mouse’s stomach	C57BL/6J mice	Liu et al. (2018)
Polydextran aldehyde (PDA), branched polyethylenimine (PEI)	Cecal ligation and puncture model	C57BL/6 mice	Giano et al. (2014)
Poly (lactic-co-glycolic acid) (PLGA), poly (ethylene glycol)	Cecal intestinal anastomosis survival model	C57BL6/J mice	Behrens et al. (2015)
Methacryloyl-substituted tropoelastin (MeTro)	Standard incision (15 mm × 15 mm × 1 mm) was generated on the lung with a scalpel	Yorkshire pigs	Annabi et al. (2017)
Gelatin	Pleural defects in *ex vivo* and *in vivo* porcine models	Pigs	Elvin et al. (2010)
Methacrylated gelatin (GelMA)	A standardized lung lobe incision (3 mm in length; 5 mm in depth toward the hilum) was generated	Male Wistar rats	Assmann et al. (2017)
Methacrylated gelatin (GelMA)	Standardized visceral pleural defect (15 mm in length; 15 mm in width; 1 mm in depth) was generated	Pigs	Assmann et al. (2017)
Gelatin, dopamine-conjugate gelatin (GelDA)	A small (3 mm) incision was created in the murine small bowel; a surgical incision (2–4 mm) was made in one of the uterine horns	C57/BL6 mice	Hong et al. (2016)
Polyvinyl alcohol (PVA), poly (acrylic acid) (PAA), N-hydroxysuccinimide (NHS) ester, sodium bicarbonate (SBC), glutathione	A laceration was made on a porcine lung lobe with a razor blade (3 cm in length); the air was applied through the tubing connected to the upper part of the trachea (25-mmHg pressure) to visualize air leakage with or without bioadhesive	Pig	Chen et al. (2020)

### Bleeding

In this review, bleeding is considered as the leakage of the blood, resulting from trauma, surgical process, diseases, and even some medicines. It is one of the most frequent complications in patients. There are many sealants available in the market for hemostasis, such as Tisseel® (Fibrin sealant), Coseal® (PEG sealant), and Bioglue® (Albumin and Glutaraldehyde). However, they have separate limits. In their indications, Tisseel® is not suggested for massive bleeding; Coseal® and Bioglue® are suggested to be used as adjunctions to sutures or staples. So, sealants with high efficacy are still highly desired for uncontrollable or massive bleeding. Different strategies have been used to develop bioadhesives for hemostasis. Cui et al. developed a hyperbranched polymer sealant with a hydrophobic backbone and hydrophilic adhesive catechol side. By introducing long alkylamine chain into the structure, their sealant showed efficient hemostasis in the rat’s femoral artery bleeding and liver bleeding model (Cui et al., 2019). In our previous work, the concept of fabricating sealants with strong cohesion strength has been used (Bu et al., 2016; Bu et al., 2019). Tough sealants based on ammonolysis-based Tetra-PEG hydrogels were fabricated, which showed promising efficacy in pigskin massive bleeding and rabbit femoral artery section models (Bu et al., 2016). Hemostasis is another critical situation for patients with coagulation disorders, such as hemophilia, Von Willebrand disease, and aged patients taking anticoagulation drugs. Shin et al. presented a hemostatic hypodermic needle that will be able to prevent bleeding following tissue puncture. The surface of the needle was coated with catechol-functionalized chitosan that would be transformed from the solid to the gel phase *in situ* to seal punctured tissues (Shin et al., 2016). Later, Kim et al. used the catechol-conjugated chitosan to fabricate a hemostatic sponge (Kim et al., 2021). They used preclinical models to evaluate the hemostatic efficacy, including the heparinized rabbit model of femoral artery bleeding, the pig model of traumatic blunt liver injury with hemodilutional and hypothermic coagulopathy, and the anticoagulant-treated rabbit model of liver resection bleeding. A further clinical study performed on 15 patients showed that this sponge demonstrated an excellent hemostatic effect compared with the commercialized controls.

### Other Leakages

Except for blood leakage, there are also some other leakage types. In lung surgery, prolonged air leakage is the most common complication after surgical dissection and resection. The criteria of an ideal sealant for lung leakage include the following: 1. The sealant can stand higher burst pressure than that generated during physiological breathing; 2. the sealant should be elastic with a proper elastic modulus to support the inflation and deflation of lung tissue. Annabi et al. used methacryloyl-substituted tropoelastin (MeTro) to engineer a highly flexible sealant (Assmann et al., 2017). After applying MeTro to a porcine model, it was found that the sealant completely sealed the severely leaking lung tissue in the absence of sutures or staples. Urinary fistulas have been considered a severe socioeconomic problem, which occurs most commonly as a result of prolonged obstructed labor, which causes pelvic floor ischemia and, at times, substantial tissue loss (Margules and Rovner, 2019). Kim et al. developed water-immiscible mussel protein–based bioadhesive, which successfully sealed *ex vivo* urinary fistulas and provided good durability and high compliance (Kim et al., 2015). Liu et al. developed gelatin methacrylate–based double-network hydrogel to manage the leakage of gastric contents without sutures successfully (Liu et al., 2018).

## Immobilization

The last category for bioadhesives includes those for immobilization (Table 3). Because of the intrinsic adhesion property, they can immobilize themselves as functional wound dressing or delivery vehicles. By adhering items together, they even can fix other medical devices.

**TABLE 3:** Bioadhesives for immobilization.

Materials used	Type of the model	Animal species	References
Functional wound dressings-skin defects			
Quaternized chitosan (QCS), benzaldehyde-terminated Pluronic®F127 (PF127-CHO)	About 1 cm diameter full-thickness round skin wounds were created by a needle biopsy	Female Kunming mice	Qu et al. (2018)
Quaternized chitosan-g-polyaniline (QCSP), benzaldehyde group functionalized poly (ethylene glycol)-co-poly (glycerol sebacate) (PEGS FA)	7 mm diameter full-thickness round skin wounds were created by a needle biopsy	Female Kunming mice	Zhao et al. (2017)
Hyaluronic acid-graft-dopamine (HA-DA), Reduced graphene oxide (rGO), polydopamine	7 mm diameter full-thickness round skin wounds were created by a needle biopsy	Female Kunming mice	Liang et al. (2019)
Poly (N-isopropyl acrylamide) (PNIPAm), alginate, chitosan	A full-thickness dorsal excisional skin wound was created on the mice with a sterile 8-mm-diameter biopsy punch following the removal of hair	Female C57BL/6J mice	Blacklow et al. (2019)
Skin secretion of *Andrias davidianus* (SSAD)	A disposable biopsy punch was used to create a full-thickness round skin wound (diameter = 10 mm) on the back	Streptozotocin-induced diabetic SD rat	Deng et al. (2019)
Polydopamine-clay-polyacrylamide (PDA-clay-PAM) hydrogel	Full-thickness skin wounds were created on the dorsal area of the rats	Male SD rats	Han et al. (2017)
Polydopamine–polyacrylamide (PDA–PAM) hydrogel	Four full-thickness circular wounds (5 mm in diameter) were created on the upper back of each mouse by a disposable 5 mm skin biopsy punch	Male SD rats	Han et al. (2017)
Ag-Lignin NPs-PAA-pectin hydrogel	Four full-thickness circular wounds (8 mm in diameter) were created on the upper back of the rats	Male SD rats	Gan et al. (2019)
Functional wound dressings-corneal defects			
Gelatin, methacrylic anhydride (MA)	A 3-mm biopsy punch was used to make a partial trephination (cut) in the central cornea of the right eye to a depth of approximately 50%	Male New Zealand white rabbits	Shirzaei Sani et al. (2019)
Functional wound dressings-cartilage defects			
Polydopamine-chondroitin sulfate-polyacrylamide (PDA-CS-PAM) hydrogel	A full-thickness defect (diameter: 3.5 mm; thickness: 5 mm) was created through the articular cartilage and subchondral bone of the patellar groove in the right leg of the rabbits using an electric drill	Japanese white rabbits	Han et al. (2018)
Functional wound dressings-calvarial defects			
Acrylate b-cyclodextrin (Ac-b-CD), methacrylated gelatin (MeGel)	Two 5-mm-diameter craniotomy defects were created in the parietal bones of the skull on each side of the sagittal suture line	Male SD rats	Feng et al. (2016)
Functional wound dressings-myocardial infarction (MI)			
Gelatin methacryloyl (GelMA), choline-based bio-ionic liquid (Bio-IL)	Immediately after the induction of MI, the scaffolds were delivered to the surface of the left ventricle, distal to the site of MI, and photo-crosslinked for 3000 s using UV light	Balb/C mice	Walker et al. (2019)
Waxy starch	The materials were patched onto the MI site of the heart.	SD rats	Lin et al. (2019)
Drug/Cell delivery			
Methacrylated alginate (Alg-DA-MA), Gingival mesenchymal stem cells (GMSCs), HAp microparticles (MPs)	*Ex vivo*–expanded human GMSC aggregates/HAp MPs (4 × 106) were encapsulated in adhesive hydrogel and implanted subcutaneously (0.50 ml) into the dorsal surface of a 5-month-old Beige nude XID III (nu/nu) (Harlan, United States) mice; titanium implants (ACE Surgical Supply, Brockton, MA) were used to introduce a well-characterized strain of A. actinomycetemcomitans biofilm transmucosally into rats	Beige nude XID III (nu/nu) (Harlan, United States) mice; Male and female SD rats	Hasani-Sadrabadi et al. (2020)
Poly (ethylene glycol), catechol	Islet transplantation: approximately 100 ml cPEG was applied following islet deposition directly on this tissue surface	Streptozotocin-induced diabetic mice	Brubaker et al. (2010)
HA-catechol (HA-CA) hydrogel	Hepatocyte transplantation: hepatocytes encapsulated in HA-CA hydrogel were transplanted onto the lobe of the native liver or liver with partial hepatectomy of athymic mice using a pipette; HA-CA hydrogel was painted onto the infarction site immediately after induction of hydrogel crosslinking	Female athymic mice (Balb/cnu); Male Hsd RH-rnu rats with myocardial infarction	Shin et al. (2015)
Tetra-PEG/agar hydrogel (PA)	The drug containing hydrogel was formed *in situ* on the surface of the rats’ skin	SD rats	Zhang et al. (2019)
Tannic acid, poly (ethylene glycol)	Each mouse was fed on 0.02 cc of the ICG-encapsulated TAPE–OH for the adhesion to the esophagus without any anesthesia	BALBc nude mice	(Kim et al., 2015; Shin et al., 2016)
Wheat germ agglutinin (WGA)-conjugated liposomes (WGA-liposomes)	The OKF6/TERT-2 cell suspension (1 × 104 cells) was seeded onto poly-d-lysine coated glass bottom micro-well dishes (MatTek Corporation) and allowed to grow in cell culture media for 24 h. WGA-conjugated CFPE liposomes (WGA-CFPE-liposomes) were added to the micro-well dishes (45 μg/ml lipid) and incubated at 37 °C for 2 h	OKF6/TERT-2 cell	Wijetunge et al. (2018)
GO (graphene oxide) hybrid supramolecular hydrogels (GO–HSH)	DOX-loaded GO–HSH hydrogel coating on titanium substrate and drug release to kill Hela	Hela	Chen et al. (2018)
Poly (lactic acid)-hyperbranched polyglycerol (PLA-HPG), camptothecin (CPT)	PDVC57 cells were harvested, washed, and resuspended, then injected into the dorsal right flank. Tumors were injected with BNP-CPT and visualized particle distribution *via* confocal microscope 72 h after injection	Wild-type C57BL/6J mice	Hu et al. (2021)
Medical device fixation			
GO (graphene oxide)-PVA (poly (vinyl alcohol)) hydrogel, GO (graphene oxide)-PVA (poly (vinyl alcohol) PAA (poly (acrylic acid)) -NHS(N-hydroxysuccinimide) ester hydrogel	The heart was exposed *via* a thoratomy, bioadhesive electrodes were used to record epicardial ECG.	Female Sprague–Dawley rats.	Deng et al. (2021)
	A circuit with light emitting diodes (LEDs) was applied to the *ex vivo* porcine heart (by introducing cyclical, pressurized air inputs into the heart chambers to mimic heartbeats) to test if electrical communication was stable enough.	*Ex vivo* porcine heart	

### Functional Wound Dressings

Advanced fixation methods are still in need because traditional wound dressing methods lack the ability of adhesion to wounds, which increases the operative difficulty index for both the patients and doctors. Compared with these methods, bioadhesives can easily fix themselves on the wound area, contributing to the increasing popularity of bioadhesives to be used as a functional wound dressing (Liang et al., 2019; Zhao et al., 2017; Blacklow et al., 2019; Han et al., 2017; Han et al., 2017; Gan et al., 2019). They are favorite candidates for skin damage, one of the most common physical injuries in human history. Based on quaternized chitosan (QCS) and benzaldehyde-terminated Pluronic®F127, Qu et al. developed antibacterial bioadhesives with rapid self-healing, extensibility, and compressibility for joints and skin wound healing (Qu et al., 2018). They loaded curcumin into the bioadhesive and found that it significantly accelerated wound healing with a higher granulation tissue thickness in a full-thickness skin defect model. Inspired by embryonic wound closure, Blacklow et al. fabricated mechanically active dressings to accelerate wound healing (Blacklow et al., 2019). The bioadhesive dressing will contract at body temperature, which further applies force to draw the wound edges together in a purse-string–like manner. Adhesive dressings are beneficial in places where the fixation is difficult, like brittle tissues. Lin et al. developed a viscoelastic adhesive patch that accommodates the cyclic deformation of the myocardium. It was found that the patch outperformed most existing acellular epicardial patches in reversing left ventricular remodeling and restoring heart function after both acute and subacute myocardial infarctions in rats (Lin et al., 2019). In addition to the heart, defects from the corneal, cartilage, and calvarial were explored to be treated with bioadhesives with good outcomes (Feng et al., 2016; Han et al., 2018; Lin et al., 2019; Shirzaei Sani et al., 2019).

#### Delivery Systems

Compared with the traditional hydrogel delivery system, the advantage of bioadhesives in delivery is that they can fix delivered items on the site. Mucoadhesion is very useful in increasing the bioavailability of poorly absorbed drugs by prolonging the residence time in the gastrointestinal tract, leading to reduced dose and dosing frequency (Han et al., 2012; Gong et al., 2017). A lot of mucoadhesive-based delivery systems were developed with some well-summarized reviews (Reddy et al., 2011; Zhang et al., 2016; Zhang et al., 2020; Pathak and Malviya, 2020). Hu et al. encapsulated camptothecin into poly(lactic acid)-hyperbranched polyglycerol-based nano-bioadhesive particles (NPs). Because of the strong bonding of these NPs to squamous cell carcinoma tumor cells, the system significantly reduced the tumor burden and enhanced survival (Hu et al., 2021). Except for the nano/micro scale mucoadhesion, macro-bioadhesives have also been developed to load drugs to achieve better healing efficacy (Zhang et al., 2019; Bu et al., 2020). Cells can also be loaded into the bioadhesives. The use of an appropriate scaffold biomaterial as a cell delivery vehicle can provide a suitable microenvironment to prolong cell viability and present essential factors to direct cell differentiation toward the desired lineages (Khademhosseini and Langer, 2016). Currently, however, a major drawback of the reported cell-laden hydrogels is the weak adhesion to the host tissue at the defective site. Hasani-Sadrabadi et al. used alginate-based photo-crosslinkable bioadhesives to load mesenchymal stem cells. It was found that the cell-loaded adhesive system leads to complete bone regeneration around the ailing dental implants with peri-implant bone loss (Hasani-Sadrabadi et al., 2020).

#### Fixation of Other Medical Devices

Nowadays, a growing interest is centered on implantable and wearable medical devices with excellent translational potential in the clinic, like tissue scaffolds, biosensors, and biodetectors. However, it is crucial to establish conformal and stable contact between those devices and the target tissue (Schiavone and Lacour, 2019; Yuk et al., 2019). Wires and sutures are required for this fixation, which raises concerns of infection, secondary tissue injury, and scaffold damage. As a noninvasive adhesion method, bioadhesives have the potential to replace these invasive fixation methods. Based on a thin layer of a graphene nanocomposite, Deng et al. developed an electrical bioadhesive that can provide rapid, robust, and on-demand detachable integration of bioelectronic devices on diverse wet dynamic tissues (Deng et al., 2021). Later, they successfully used the e-bioadhesive to record an *in situ* epicardial electrocardiogram and electrically stimulated a sciatic nerve on a rat model. This technique offers a promising solution for addressing the long-standing challenges in tissue–device integration. Another good aspect of bioadhesives to be used in these situations is that different functions can be added into the bioadhesives to improve the outcome of the medical devices or reduce the potential complications. For example, the antibacterial property can be introduced to reduce the chance of medical devices’ infection (Hwang et al., 2018). In fact, there is still a vast area of bioadhesives in medical device fixation waiting to be explored. However, one should be careful because the bioadhesives may also adversely influence the medical devices. Macnab et al. showed that Tisseel® significantly attenuated NIR light of a near-infrared spectroscopy during *in vitro* transmittance and critically compromised photo transmission *in vivo* (Macnab et al., 2018). Another fixation method is also required when there is a need for tissue transplantation. Islet transplantation is used to treat type I diabetes by replacing the lost beta cell function. Brubaker et al. directly immobilized islets onto intra-abdominal tissue surfaces using a thin layer of a mussel-inspired bioadhesive (Brubaker et al., 2010). On the one hand, the fixation approach offers the potential advantages for convenient, rapid, and minimally invasive islet transplantation by direct apposition of the islet bolus onto tissue surfaces. On the other hand, the technique avoids the intravascular engraftment site, eliminating adverse effects of first-pass blood exposure in the liver while maintaining the capability of rapid islet revascularization and the benefits of direct insulin secretion into the portal circulation.

## Perspective

Bioadhesives are believed to revolutionize the surgical process (Mehdizadeh and Yang, 2013; Taboada et al., 2020). They have already been widely used as adhesives and sealants in the clinic to reduce complications and improve outcomes. However, those commercialized products are still far from satisfactory. Fibrin-based, PEG-based, and cyanoacrylate-based bioadhesives are the most commonly used ones. Fibrin-based and PEG-based bioadhesives have good biocompatibility but weak adhesion strength. So, most of them are only used as adjunctions for traditional wound closure or sealing methods. Cyanoacrylate-based bioadhesives have strong adhesion strength, but their potential safety concerns limit their wide applications, especially internal applications. Thus, more powerful and commercially transformable bioadhesives for wound closure and sealing leakage are still needed.

Compared with traditional wound dressings, bioadhesives get easily attached to the parts where they are applied because of their intrinsic adhesion property (Li and Mooney., 2016). So, there is a growing interest in using bioadhesives as a functional wound dressing. This application is beneficial for tissues where the fixation of traditional wound dressing fails to work, like a beating heart and brittle brain (Lin et al., 2019). However, the absence of removability makes it hard for further wound care or dressing change, resulting in more potential troubles when mechanical debridement is involved. So controllably removable property is also explored for bioadhesives (Chen et al., 2020; Bu et al., 2019; Villa-Camacho et al., 2015; Konieczynska et al., 2016).

Using bioadhesives for the local delivery of functional items like drugs or cells is also a promising way to realize specialized and prolonged effectiveness. Compared with conventional hydrogel vehicles, bioadhesives can adhere to tissues, making them more stable in special tissues like the beating heart and esophagus (Lin et al., 2019). By mixing Tannic and PEG, Lee et al. developed a new medical glue called TAPE, which had been applied to the esophagus and demonstrated the ability to detect gastroesophageal reflux diseases because it maintained wet-adhesive properties (Kim et al., 2015). Bioadhesives are also used to fix medical devices or tissues, of which the importance is increasing with an increasing number of implantable medical devices and tissue transplantation. The fixation using bioadhesives will not damage either medical devices or the tissues. Although very promising, there is a difficulty in avoiding the interference between the functions of medical devices and bioadhesives. Besides, for tissue transplantation, the adhesion strength of bioadhesives available might not be sufficient for large pieces of tissues.

Although massive efforts have been spent on developing bioadhesives, there are only a handful of products available in the market (Taboada et al., 2020). First, the researcher might care too much about the adhesion mechanism, while cohesion is ignored. Cohesion dramatically influences how the bioadhesives would be used, which is particularly important for clinical translation. In the market, ease of use has a positive influence on people’s choices. Second, one bioadhesive never fits all the applications. The requirement of bioadhesives for wound closure differs from those for sealing leakage. So, it is suggested to choose the unmet clinical target first and then the relative characterization methods to fabricate bioadhesives for translation.
